# Supplementary material for: Resolving the full spectrum of human genome variation using Linked-Reads
Source: Genome Res. 2019 Apr;29(4):635–45. doi: 10.1101/gr.234443.118 (PMC6442396; doi:10.1101/gr.234443.118)
Supplement: Supplemental Material [file supp_gr.234443.118_Supplemental_longranger-2.2.zip › longranger-2.2.tar.bz2!/lr2.2-public/lib/stan/cmdstan/stan/doc/api/html/agrad_2rev_2functions_2log__sum__exp_8hpp_source.html]

Stan: src/stan/agrad/rev/functions/log\_sum\_exp.hpp Source File


|  |  |
| --- | --- |
|  | Stan  2.5.0  probability, sampling & optimization |


- Main Page
- Related Pages
- Namespaces
- Classes
- Files

- File List
- File Members

All Classes Namespaces Files Functions Variables Typedefs Enumerations Enumerator Friends Macros Pages

- src
- stan
- agrad
- rev
- functions

log\_sum\_exp.hpp

Go to the documentation of this file.

1 #ifndef STAN\_\_AGRAD\_\_REV\_\_FUNCTIONS\_\_LOG\_SUM\_EXP\_HPP

2 #define STAN\_\_AGRAD\_\_REV\_\_FUNCTIONS\_\_LOG\_SUM\_EXP\_HPP

3

4 #include <stan/agrad/rev/var.hpp>

5 #include <stan/agrad/rev/calculate\_chain.hpp>

6 #include <stan/agrad/rev/internal/vv\_vari.hpp>

7 #include <stan/agrad/rev/internal/vd\_vari.hpp>

8 #include <stan/agrad/rev/internal/dv\_vari.hpp>

9 #include <stan/agrad/rev/internal/vector\_vari.hpp>

10 #include <stan/agrad/rev/operators/operator\_greater\_than.hpp>

11 #include <stan/agrad/rev/operators/operator\_not\_equal.hpp>

12 #include <stan/math/functions/log\_sum\_exp.hpp>

13

14 namespace stan {

15  namespace agrad {

16

17  namespace {

18  double log\_sum\_exp\_as\_double(const std::vector<var>& x) {

19  using std::numeric\_limits;

20  using std::exp;

21  using std::log;

22  double max = -numeric\_limits<double>::infinity();

23  for (size\_t i = 0; i < x.size(); ++i)

24  if (x[i] > max)

25  max = x[i].val();

26  double sum = 0.0;

27  for (size\_t i = 0; i < x.size(); ++i)

28  if (x[i] != -numeric\_limits<double>::infinity())

29  sum += exp(x[i].val() - max);

30  return max + log(sum);

31  }

32

33  class log\_sum\_exp\_vv\_vari : public op\_vv\_vari {

34  public:

35  log\_sum\_exp\_vv\_vari(vari\* avi, vari\* bvi) :

36  op\_vv\_vari(stan::math::log\_sum\_exp(avi->val\_, bvi->val\_),

37  avi, bvi) {

38  }

39  void chain() {

40  avi\_->adj\_ += adj\_ \* calculate\_chain(avi\_->val\_, val\_);

41  bvi\_->adj\_ += adj\_ \* calculate\_chain(bvi\_->val\_, val\_);

42  }

43  };

44  class log\_sum\_exp\_vd\_vari : public op\_vd\_vari {

45  public:

46  log\_sum\_exp\_vd\_vari(vari\* avi, double b) :

47  op\_vd\_vari(stan::math::log\_sum\_exp(avi->val\_, b),

48  avi, b) {

49  }

50  void chain() {

51  avi\_->adj\_ += adj\_ \* calculate\_chain(avi\_->val\_, val\_);

52  }

53  };

54  class log\_sum\_exp\_dv\_vari : public op\_dv\_vari {

55  public:

56  log\_sum\_exp\_dv\_vari(double a, vari\* bvi) :

57  op\_dv\_vari(stan::math::log\_sum\_exp(a, bvi->val\_),

58  a, bvi) {

59  }

60  void chain() {

61  bvi\_->adj\_ += adj\_ \* calculate\_chain(bvi\_->val\_, val\_);

62  }

63  };

64

65  class log\_sum\_exp\_vector\_vari : public op\_vector\_vari {

66  public:

67  log\_sum\_exp\_vector\_vari(const std::vector<var>& x) :

68  op\_vector\_vari(log\_sum\_exp\_as\_double(x), x) {

69  }

70  void chain() {

71  for (size\_t i = 0; i < size\_; ++i) {

72  vis\_[i]->adj\_ += adj\_ \* calculate\_chain(vis\_[i]->val\_, val\_);

73  }

74  }

75  };

76  }

77

81  inline var log\_sum\_exp(const stan::agrad::var& a,

82  const stan::agrad::var& b) {

83  return var(new log\_sum\_exp\_vv\_vari(a.vi\_, b.vi\_));

84  }

88  inline var log\_sum\_exp(const stan::agrad::var& a,

89  const double& b) {

90  return var(new log\_sum\_exp\_vd\_vari(a.vi\_, b));

91  }

95  inline var log\_sum\_exp(const double& a,

96  const stan::agrad::var& b) {

97  return var(new log\_sum\_exp\_dv\_vari(a, b.vi\_));

98  }

102  inline var log\_sum\_exp(const std::vector<var>& x) {

103  return var(new log\_sum\_exp\_vector\_vari(x));

104  }

105

106  }

107 }

108 #endif

log\_sum\_exp.hpp

dv\_vari.hpp

stan::agrad::log\_sum\_exp

fvar< T > log\_sum\_exp(const fvar< T > &x1, const fvar< T > &x2)

**Definition:** log\_sum\_exp.hpp:15

operator\_greater\_than.hpp

stan::math::max

double max(const double a, const double b)

**Definition:** max.hpp:7

vd\_vari.hpp

stan::agrad::sum

fvar< T > sum(const Eigen::Matrix< fvar< T >, R, C > &m)

**Definition:** sum.hpp:14

stan::agrad::var::vi\_

vari \* vi\_

Pointer to the implementation of this variable.

**Definition:** var.hpp:40

vector\_vari.hpp

operator\_not\_equal.hpp

var.hpp

stan::agrad::calculate\_chain

double calculate\_chain(const double &x, const double &val)

**Definition:** calculate\_chain.hpp:8

stan::agrad::var

Independent (input) and dependent (output) variables for gradients.

**Definition:** var.hpp:27

calculate\_chain.hpp

size\_

size\_t size\_

**Definition:** dot\_self.hpp:18

vv\_vari.hpp

stan::agrad::log

fvar< T > log(const fvar< T > &x)

**Definition:** log.hpp:15

stan::agrad::exp

fvar< T > exp(const fvar< T > &x)

**Definition:** exp.hpp:16


---

[ Stan Home Page ]

*© 2011–2014,
Stan Development Team.*
